# Hepatitis C Virus: The Rising Concerns and Growing Hopes, Report From the HCV Symposium, Fourth Tehran Hepatitis Congress, November 2011, Tehran, Iran

**DOI:** 10.5812/hepatmon.6679

**Published:** 2012-07-30

**Authors:** Seyed Moayed Alavian, Hossain Jabbari, Nasser Ebrahimi Daryani, Mohammad Torabi nami

**Affiliations:** 1 Baqiyatallah Research Center for Gastroenterology and Liver Disease, Baqiyatallah University of Medical Sciences, Tehran, IR Iran; 2 Digestive Disease Research Institute (DDRI), Infectious Diseases Department, Shariati Hospital, Tehran University of Medical Sciences, Tehran, IR Iran; 3 Vienna General Hospital (AKH), Univ. Department of Dermatology, Department of Clinical Immunology, Allergy and Infectious Diseases, Wien, Austria; 4 Departments of Gastroenterology and Hepatology, Tehran University of Medical Sciences, Tehran, IR Iran; 5 Department of neuroscience, Institute for Cognitive Science Studies, Shahid Beheshti Medical University, Tehran, IR Iran

**Keywords:** Hepatitis C, Congresses, Iran

## Abstract

The rising concerns for future health burden of hepatitis C virus (HCV) in global scale has continuously encouraged preventing measures particularly public awareness programs. There is an increasing necessity for allocating HCV awareness issues in public scope, especially for high risk populations and patients. Proper knowledge of health care professionals and treating physicians and their attitude with regard to hepatitis C management is also crucial. Achieving this can be a constructive step forward in controlling and hopefully eradicating hepatitis C virus in our community. Having a clear scientific grasp on treatment options and protocols, the concept of “CURE” achievement in hepatitis C and the future hopes in enhancing virological response with the coming direct antiviral agents can significantly add to the current practices of treating hepatitis C. This scientific report paper outlines the insights communicated at the HCV symposium during the 4th Tehran Hepatitis Congress, November 2011, Tehran, Iran.

## 1. Introduction

The 4th Tehran Hepatitis Congress (THC4) as an international forum for presenting “state-of-the-art” and cutting edge updates in clinical care of viral hepatitis basic researches, was held from the 23rd to 26th of November, 2011 in Tehran, Iran. During the first day of the event a well-focused symposium “HCV; the rising concerns and growing hopes” was conducted. The global and local burden of HCV, treatment updates and the concept of cure achievement in hepatitis C treatment were detailed and discussed. This article tries to outline main issues and insights, which were communicated during the above scientific session.

## 2. Public Health Implications of HCV; the Rising Concerns and Growing Hopes for HCV Eradication

### 2.1. The Global and Local Image of Hepatitis C

Hepatitis C is considered as a major cause of liver-related morbidities and mortalities worldwide. Global records and estimations show that the occurrence of end stage liver disease caused by HCV (Cirrhosis, fibrosis, Hepatocellular carcinoma and) will likely peak around the year 2020. Today, there are around 170-200 million individuals living with HCV worldwide. The analyzed data reported from the United States show that HCV is seen as the etiologic factor for acute hepatitis (20%), chronic hepatitis (70%), liver cirrhosis (40%), hepatocellular carcinoma (60%) and liver transplants (40%) in a noticeable number of liver disease patients [1]. In a systematic review, HCV prevalence in general population of Iran was reported less than 1%. Tehran and Guilan provinces with the prevalences of 1.3 % and 0-0.16 % respectively were considered as the most and the least prevalent geographic areas for HCV [2]. Statistics show that intravenous drug use (IDU) accounts for 50% of HCV transmission routes in our country. Other less important risk factors are male gender (OR = 10), tattooing (OR = 8.1), unmarried status (OR = 6.9), living in rural areas (OR = 3.4) and history of transfusion (OR = 1.1). In the countries under the Eastern Mediterranean Regional Office of WHO, nearly 32% (95% CI: 31-33) of hemodialysis patients are currently infected with HCV [3]. In Iran, however, there are promising recent statistics with regard to specific transmission modes. In end stage renal disease patients, prevalence was 25% in a late 2009 study report, which decreased to 7.61% in the year 2010. Prevalence of HCV among spouses is 1.33% which is not higher than controls statistically. Therefore intra-familial contacts are not considered as an important route of HCV transmission. In a phylogenic study done on 125 Iranian patients, the most prevalent subtype of HCV in Iran was 1a (47%). The 3a, 1b and 4 genotypes were reported in 36%, 8% and 1% of HCV patients respectively [4].

### 2.2. The Natural History of Hepatitis C 

Following acute HCV infection, roughly 80% of patients will have the persistent infection and fail to experience recovery. Among the persistent infected patients, some will have a stable chronic hepatitis (30%), some will experience variable progression (40%) and others will progress to severe stages (30%) [5].

Of those with chronic HCV infection, 80% would remain stable, while 20% would ultimately have cirrhosis from which 5% will develop end stage of liver failure or hepatocellular carcinoma [5]. Considering the HCV prevalence and the country population of Iran, there will be over 5000 patients estimated to need liver transplantation over next 10 to 20 years.

### 2.3. Reducing the HCV Related Liver Complications 

Preventing liver injury caused by HCV is normally done in four different levels, primordial to tertiary. Primary preventions include all measures to help preventing new HCV infections to occur. Since there are no vaccines available for HCV (primary prevention), these efforts mainly focus on risk behaviors reduction (primordial prevention). This is partly done by primarily increasing public awareness and particularly alert those high risk populations about transmission routes and subsequent burden of HCV [6]. Secondary and tertiary preventions include all measures exercised to reduce complications of liver disease in those with chronic infection. This will be achieved firstly by identifying infected individuals and reversing or eliminating factors which accelerate the rate of fibrosis progression. There are therapies to stabilize or reverse liver injury and fibrosis [6][7].

### 2.4. Screening for HCV and Cirrhosis in Infected Cases

The high risk population, visited in our daily practice, is believed to have at least one of the following risk factors [8].

1. People who ever injected illegal drugs, even who did it once many years ago.

2. People with selected medical conditions including those:

a. Receiving clotting factor concentrates made before 1990.

b. On hemodialysis or “ever had it”.

c. With history of organ transplant (recipient) or transfusion before 1992.

3. Healthcare providers, emergency medical and public safety workers after needle sticks, or mucosal exposure to HCV contaminated blood or body fluids.

4. Children born to HCV positive women.

**Figure 1:**
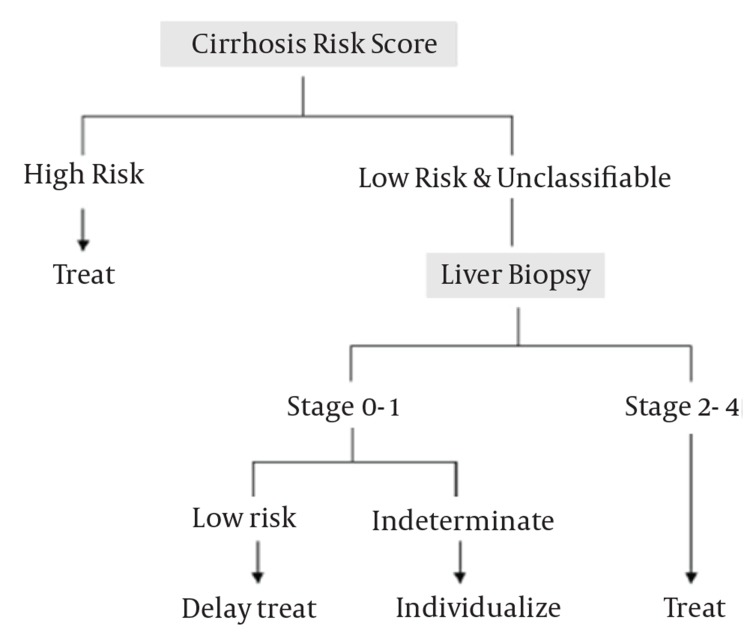
High Cirrhosis Risk Score (CRS) May Streamline Towards Treatment Before Liver Biopsy Gressner oA, Weiskirchen R, Gressner AM, Biomarkers of hepatic fibrosis, fibrogenesis and genetic pre-disposition pending between fiction and reality, Journal of Cellular and Molecular Medicine, John Wiley and Sons Ltd, with permission.

Patients who are already afflicted with hepatitis C, some have factors which predispose development of cirrhosis. These factors include, later age, male gender, ethnicity, higher body mass index, alcohol intake(> 40-50 mg/day), genotype (GT) 1, high GT quasispecies diversity, viral coinfection (HBV and HIV), cigarette smoking and being genetically prone to alpha-1 antitrypsin deficiency, use of steroids, and genetic polymorphism [8]. There is a newly developed genetic test to predict fibrosis progression probability in patients with chronic HCV. In this assay certain single nucleotide polymorphisms (SNPs) are assessed in seven genes and then the pattern of SNPs is converted to a numerical score known as CRS (Cirrhosis Risk Score) [9][10]. CRS identifies patients who will more rapidly progress to cirrhosis. Patients with quite high CRS need not undergo liver biopsy and can be considered for HCV treatment (Figure 1). This assay does not seem to be commercially available in our settings; however its prognostic value and ability to bypass liver biopsy before treatment commencement for such rapidly progressive patients should be re-emphasized. Among non-invasive tests of the same line, there are currently serum tests available which can use blood chemistry as surrogates in defining level of fibrosis. Wide application of these tests must depend on careful evaluation of their validity and reliability in the real practice [10].

### 2.5. Treatments of HCV Infection

**Figure 2:**
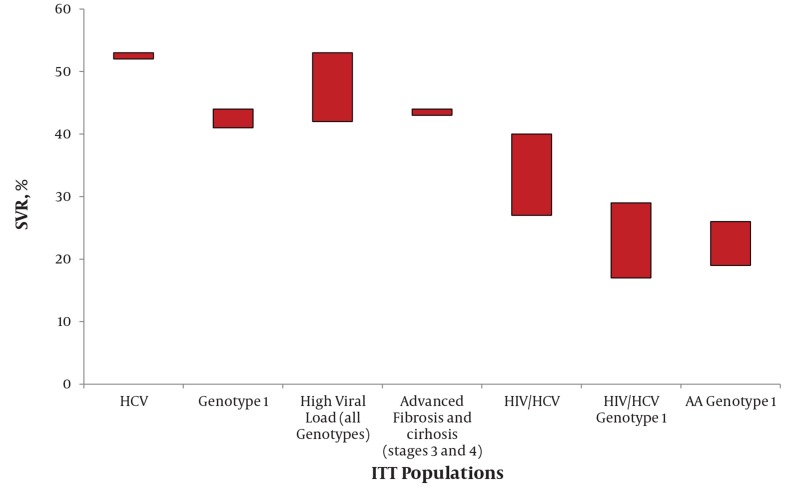
SVR Rates in HCV Infected Populations / Response Predictive Variables

Treatment regimens for chronic hepatitis C (CHC) have progressed within the past decade. The current standard of care which is the combination therapy of pegylated interferon alpha (PegIFNα) and ribavirin (RBV), has significantly improved sustained virological response (SVR) rates. There are some indicators which define and predict the response rate. The cardinal response predicting factors are GT (GT1 versus GT2 and GT3), baseline viral load (high versus low viral load in all genotypes), advanced fibrosis level (stage 3 and 4 versus stage 1 or 2) and cirrhosis. Figure 2 demonstrates how SVR achievement rate could vary in different prognostic profile patients [11][12][13][14][15][16] (Figure 2).

### 2.6. Growing Hopes for Eradication

There are two recent FDA approved agents which are available worldwide (Boceprevir and Teleprevir). Both agents belong to the first generation HCV non-structural (NS) 3/4A protease inhibitors. Availability of these agents, which have been added to the current standard of care (SOC) for specific patients, has offered a new treatment option for patients infected with HCV, who are GT 1 and have been non-responder or have relapsed to previous therapies. The comparison between the current SOC and soon available direct antivirals as protease inhibitors, namely Boceprevir and Teleprevir shows that in previously non-responders to SOC, addition of protease inhibitors (PIs) may increase SVR rate from 17-21% to 59-66% that roughly means three times more than previous response rates [17]. The triple therapy regimen is now available in US and Canada and is expected to be reachable in the Middle East including Iran in 2012. We should remain consistent with the SOC for all patients including GT1 cases and leave triple therapies (PegIFNα / RBV / Boceprevir or PegIFNα / RBV / Teleprevir) for specific patient categories which includes protease inhibitors.

## 3. On Treatment Management With Standard of Care; The Evidence for “Cure” Achievement

### 3.1. Treatment Options for HCV; the Historical Perspective

HCV life cycle is believed to be dependent on series of intra-cytosolic events in host cells. RNA is first replicated and then translated to proteins. The biochemical nature of cascade of events leading to HCV RNA replication and ultimately translation and poly protein processing is partly altered by immunologic interventions. Although interferon alpha does not seem to have direct antiviral effect on HCV, its bounding to Jack-Stat receptor is proven to stop HCV gene transcription and replication based on in-vitro and in-vivo studies [18]. Interferon alpha (IFNα) was introduced as an anti -HCV agent in 1959. HCV monotherapy with IFNα for 24 to 48 weeks resulted in 10% to 20% of virological response (1991) and this modest efficacy brought up the need for more investigations. Adding ribavirin (RBV) to IFNα (1998) resulted in improved response rates up to 30% to 40 %. Since 2000, Pegylated IFNα plus RBV has been considered as a more efficacious and preferred regimen which results in over 54% to 63% sustained virological response in various genotypes [19][20][21]. Currently there are two approved PegIFNα formulations available for the treatment of chronic hepatitis C. These include PegIFNα-2a (Pegasys®) and PegIFNα-2b (PegIntron®). PegIFNα-2a is usually given at the dose 180mcg subcutaneously once weekly (QW) and PegIFNα-2b at 1.5 mcg/kg subcutaneously once weekly. Both medications are administered together with weight based ribavirin (Copegus® or Rebetol®) at the dose of 800-1200 mg and 600-1400 mg PO daily based on HCV GT,respectively. Apart from both above mentioned PegIFNα which are readily available in Iran, there is a another Pegylated IFNα-2a known as Pegaferon® (Iranian branded with different pegylation sites) which is administered at 180 mcg SC once weekly together with RBV at 800-1200 mg PO daily. Also there are other older available medicines such as interferon alpha-2a (Roferon®), interferon alpha-2b (Intron-A®) , consensus interferon (Infergen®) and PD-feron (an Iranian branded conventional IFN) which are administered at 3-4.5 m-IU/three times weekly, 3m-IU TIW, 9mcg TIW as monotherapy, and 4.5 mega units 3thimes weekly, respectively [19]. The efficacy and safety of the two FDA approved brands of PegIFNα were evaluated and compared together in a large phase 3b randomized clinical trial engaging 118 academic centers in US and Canada. 3070 GT 1, treatment naive patients were enrolled and randomized to receive one of the following regimens. Arm1: Pegylated IFNα-2a, 180 mcg/W + 1000-1200 mg RBV/d, arm2: PegIFNα-2b, 1.5mcg/kg/W + 800-1400 mg RBV/d and arm3: PegIFNα-2b, 1mcg/kg/W + 800-1400 mg RBV/d. All patients received 48 weeks of treatment with PegIFNα and equivalent dose of RBV. The primary endpoints were SVR achievement and safety and secondary endpoints, the predictability and relapse rate [22]. Results showed that the three arms had same SVR rates (39% to 41%) and were not different in terms of adverse events. However, PegIFNα-2b regimen resulted in lower relapse and higher predictability of response. The results of this elegant study which is known as IDEAL trial [22], re-confirms what is currently stated in practice guidelines for the use of PegIFNα-2a or 2b with no specific privilege of one over the other. The results of two single arm multi-centered trials we conducted to assess the efficacy and safety of the locally manufactured PegIFNα-2a in hepatitis-C, showed the SVR of 67% for GT 1, 95% for GT 2 and 3 and 77.8% for all genotypes [23][24]. However, we wish to be able to design and run randomized clinical trials comparing the efficacy and safety of the locally manufactured PegIFNα-2a with the branded PegIFNα-2a and 2b to arrive at more interesting and dependable data in this respect.

### 3.2. Adherence to Hepatitis C Virus Therapy

**Figure 3:**
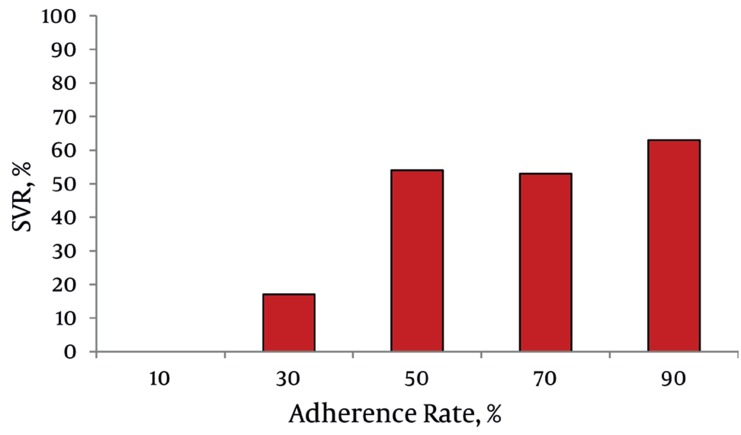
Retrospective Analysis of pegIFN alfa-2b/RBV Trials (n = 511). McHutchison JG, Manns MP, Patel K, Poynard T, Lindsay KL, Adherence to combination therapy enhances sustained response in genotype-1–infected patients with chronic hepatitis C, Gastroenterology, John Wiley and Sons Ltd, with permission

One of the main predictive factors for response to HCV treatment is the patient’s adherence to therapy. There are number of studies showing that adherence is generally high (70-98%) at the prime of the treatment journey. patients adherence is higher for PegIFNα than for RBV and decreases by time for both drugs [25]. There are issues like literacy, financial hurdles and socioeconomic problems such as improper living status which are proven to hamper patients abilities to sufficiently adhere to the prescribed treatment protocols. One of the major barriers to adherence is the side effects of the prescribed regimens. Patients often start to feel uncomfortable with these medicines so their compliance declines. The most prevalent side effects with SOC regimens include constitutional symptoms not just restricted to headache, pyrexia, myalgia, weight loss, pruritus, fatigue and depression [22]. Timely management of the occurred adverse effects seen with these medications would make patients more motivated to keep up with the therapy throughout the course. We recommend treating physicians to fully describe the treatment journey and the possible untoward effects of the prescribed medications to their hepatitis C patients. Patients are expected to remain more adherent to the suggested treatments when they know for what reason (HCV virus eradication and preventing further liver damages) they need to have all those weekly injections and daily capsules to ensure they have received whatever therapy schedule you suggest them. Refilling patients pill boxes for them, creating easy-to-follow dosing and refill schedules, filling in log books, and helping them set alarms to remind them to take their medications may all help to improve adherence. In IDEAL trial those who were adherent to the advised treatment and received at least 80% of PegIFNα dose and 80% of RBV over 80% of the treatment course experienced significant higher SVR rates (20% to30% more SVR compared to those who did not have 80/80/80 adherence) [22]. Similarly, a retrospective analysis on PegIFNα-ab/RBV treated patients reported by McHutchison JG. et al., showed a notable rise in SVR (%) when adherence rate had been improved [26] (Figure 3).

### 3.3. Goals of Treatment in HCV Infection and the Failure Scenarios

**Figure 4:**
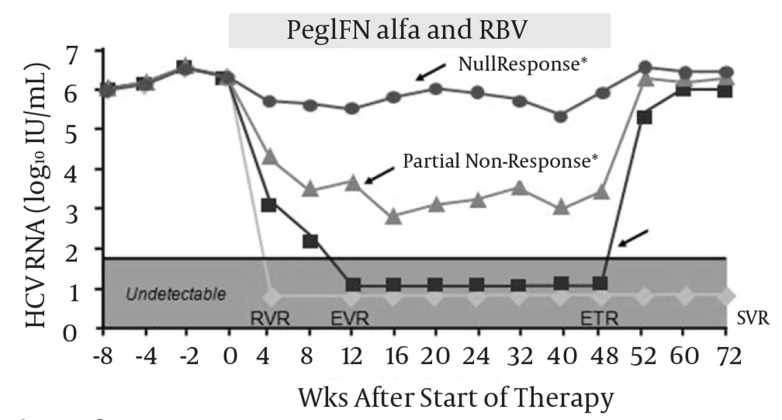
Response Criteria Definition-Patterns of Suboptimal Response. Ghany MG, et al., An Update on Treatment of Genotype 1 Chronic Hepatitis C Virus Infection: 2011 Practice Guideline by the American Association for the Study of Liver Diseases, Hepatology, John Wiley and Sons Ltd, with permission

**Figure 5:**
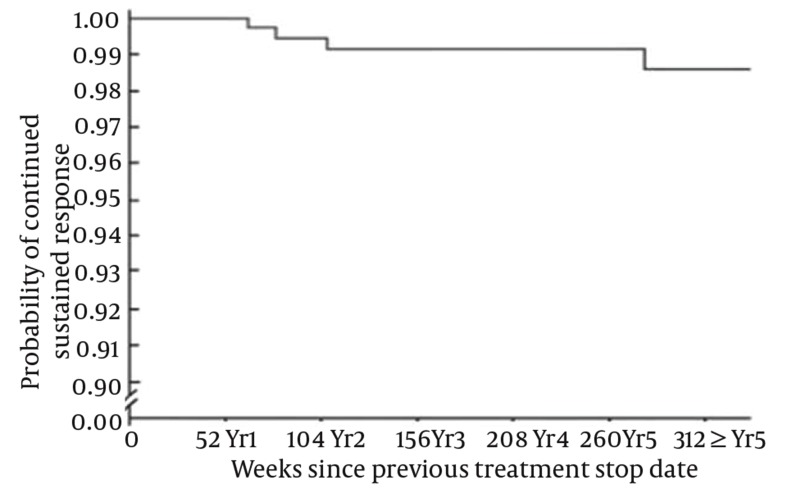
99% of the Patients Who Achieved SVR Continued to Have Negative HCV-RNA Over a five Year Follow Up Manns M, Lindsay KL, Gordon SC, et al., Sustained virological response after peginterferon alpha 2b and ribavirin predicts long-term clearance of HCV at 5 year follow up, J Hepatol, John Wiley & Sons Ltd, with permission

**Figure 6:**
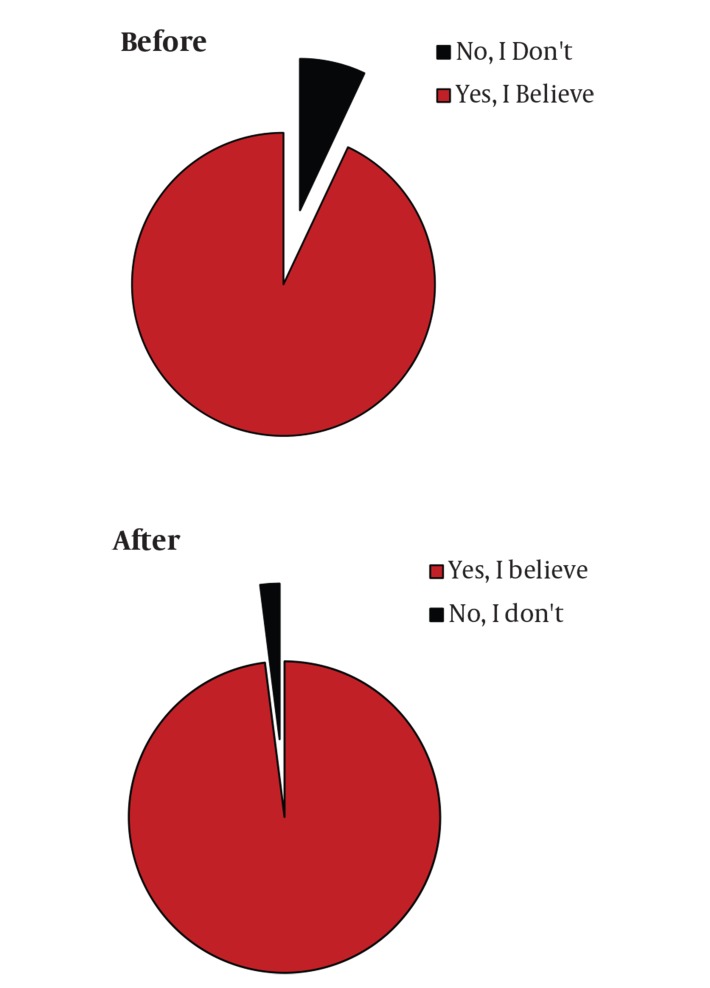
“CURE” for Hepatitis C

When treating chronic hepatitis C, the goal of treatment is to eradicate the virus, which is predicted by the achievement of an acceptable SVR. The ultimate goal is arriving at “cure” defined as remaining HCV-RNA negative for at least five years after achieving SVR. Despite using the standard of care regimens for treating of hepatitis C, there are patients who face treatment failure to antiviral therapies. Failure to treatment is categorized into different entities. These include relapse, non-response, null response, and viral breakthrough. Relapsers experience undetectable HCV RNA during treatment but have the virus reappeared after treatment is stopped. Non-responders on the other hand fail to have cleared HCV RNA from their sera after 24 weeks of therapy. The null response to therapy means failure to have decreased HCV RNA by 2 logs after 24 weeks of therapy. And finally there are patients who are categorized to have viral breakthrough. In these patients HCV RNA reemerges after it becomes undetectable while on treatment [27][28] (Figure 4). When patients achieve the SVR, this raises the question whether the achieved response is sustained for following years or not. The longer the time with HCV RNA negative after SVR, the lower the risk of recurrence, and this is the key factor for the concept of “cure”. With regard to hepatitis C, the concept of cure achievement is defined under multifaceted response criteria [27]. The subcategories of response criteria which may define “cure” achievement in HCV patients include virological, clinical, immunological, biochemical and histological responses, of which virological response is crucial. Virological response leading to cure is defined as durability of the SVR over a long term follow up (i.e. five years). SVR rate per se, depends on treatment response criteria during the treatment course and specific milestones. Early virologic response (EVR) is when at least a 2 log reduction in HCV RNA (a partial EVR) or HCV RNA negativity (a complete EVR) occurs by week 12 of treatment. End of treatment response (ETR) is defined as HCV RNA negativity at the end of treatment. Rapid virologic response (RVR) is when HCV RNA negativity occurs after four weeks of treatment and delayed virologic response means HCV RNA positivity at week 12 but negative by week 24 [28] (Figure 4). Today we have evidence denoting that, SVR could be durable and almost all patients who have arrived at SVR should be considered as cured. From the evidence for cure achievement there are two reports by Mann M. et al. [29] and Swain MG et al. [19], which suggest that SVR achievement by PegIFNα 2 b or 2a, would lead to almost 99% sustainability of response in a five year follow up. There are only less than 1% of patients who have relapsed after SVR. Based on these data, SVR (24 weeks after therapy with PegIFNα ± RBV) predicts long-term clearance of HCV. Consequently successful treatment (i.e. SVR) of hepatitis-C with PegIFN α (± RBV) leads to clinical cure of this chronic disease (Figure 5). At this symposium, we evaluated the attitude of the participants by asking them the question whether they believe there is a realistic concept of cure in hepatitis C management. After asking the question, participants either raised the red (NO) or green (Yes) cards they were already provided with. Their pre- and post-communication attitude with regard to hepatitis C cure concept is illustrated in (Figure 6).

## 4. Conclusion 

This communication in a form of a dedicated HCV symposium during the 4th Tehran Hepatitis Congress (THC4) November 2011 in Tehran, Iran aimed at shedding light to some cardinal issues with regard to current practices in the field of hepatitis C: public health implications of HCV; the rising concerns and growing hopes for HCV eradication, treatment management with the standard of care approach; the evidence for “CURE” achievement, and the future for HCV therapy were the main topics discussed during the event. Pre and post communication, assessment of the participants’ attitude with regard to the concept of “CURE” achievement in hepatitis C was done, suggesting the effectiveness of the activity. Conduction of such a kind scientific symposia with the contribution of expert panels should be even more encouraged in regular basis.
